# Automatic Controversy Detection Based on Heterogeneous Signed Attributed Network and Deep Dual-Layer Self-Supervised Community Analysis

**DOI:** 10.3390/e27050473

**Published:** 2025-04-27

**Authors:** Ying Li, Xiao Zhang, Yu Liang, Qianqian Li

**Affiliations:** 1 Key Laboratory of Symbolic Computation and Knowledge Engineering, College of Computer Science and Technology, Jilin University, Changchun 130012, China; liying@jlu.edu.cn (Y.L.); xiaozhang21@mail.jlu.edu.cn (X.Z.); liangyu21@mails.jlu.edu.cn (Y.L.); 2 Institutes of Science and Development, Chinese Academy of Sciences, Beijing 100190, China

**Keywords:** controversy, heterogeneous signed attributed network, semantic information, topology information

## Abstract

In this study, we propose a computational approach that applies text mining and deep learning to conduct controversy detection on social media platforms. Unlike previous research, our method integrates multidimensional and heterogeneous information from social media into a heterogeneous signed attributed network, encompassing various users’ attributes, semantic information, and structural heterogeneity. We introduce a deep dual-layer self-supervised algorithm for community detection and analyze controversy within this network. A novel controversy metric is devised by considering three dimensions of controversy: community distinctions, betweenness centrality, and user representations. A comparison between our method and other classical controversy measures such as Random Walk, Biased Random Walk (BRW), BCC, EC, GMCK, MBLB, and community-based methods reveals that our model consistently produces more stable and accurate controversy scores. Additionally, we calculated the level of controversy and computed *p*-values for the detected communities on our crawled dataset Weibo, including #Microblog (3792), #Comment (45,741), #Retweet (36,126), and #User (61,327). Overall, our model had a comprehensive and nuanced understanding of controversy on social media platforms. To facilitate its use, we have developed a user-friendly web server.

## 1. Introduction

With the popularity of social media, individuals now have the freedom to express their opinion and engage in hot events which often spark intense debates, leading to increased polarization and controversy. Controversy is a social phenomenon that unfolds around specific issues, characterized by the public expression and interaction of differing stances. This interaction typically manifests as polarized positions and may be accompanied by sustained debates, criticisms, or even conflicts. We can use controversy analysis to study the opposition and conflicts of different viewpoints in fields such as society, politics, and culture, and reveal the roots and impacts of controversies. Social media provides a window for researchers to analyze controversy: studying controversial hot events on the Internet has a broader impact and enables us to gain a deeper understanding of the social and public perceptions of these hot events, as well as promptly capture public opinion to safeguard it. Researchers have previously analyzed U.S. elections [1]. Democrats and Republicans are considered as two distinct communities for studying controversial phenomena on Twitter. Furthermore, recommendations on social media have a significant influence on users by propagating information [2], which can potentially exacerbate the controversies surrounding hot events in society.

The field of controversy analysis has emerged and gained significant attention, becoming a prominent area of research in recent years. According to the “spiral of silence” theory, individuals will first assess the “opinion climate” they are in before expressing their opinions: if they perceive that their own opinions belong to the mainstream, they tend to voice their views actively; if they believe that their opinions are in the minority, they may choose to remain silent. And analyzing the interaction relationships between different communities is precisely the key to assessing the degree of controversy. Many studies have tried to identify the controversial hot event from large volume of user-generated data [3]. The existing methods of controversy analysis can be summarized into three categories: content-based [4,5], network-based [6,7], and combined models [8,9]. The content-based methods take into consideration content information and utilize natural language processing methods to process the content data. To evaluate the controversial nature of the content, specific words and sentences are assigned scores or labels indicating their level of controversy. In network-based methods, they analyze the relationships and connections between users within a network to identify and understand controversial hot event interactions. By studying the topology of the network, researchers can uncover patterns and structures that indicate the presence of controversies. Numerous studies have revealed the polar structure of controversial events. These findings [10] highlighted the presence of opposing factions or perspectives within controversial hot events and researchers have successfully identified social groups in conflict by using the method of matrix factorization. However, these approaches may have limitations in capturing the nuanced aspects of controversies, as they only focus on conversation interaction, overlooking the content exchanged within the network. To address this limitation, researchers have recognized the value of incorporating content information alongside the network structure analysis. In the combined models, researchers achieve improved results in understanding controversies, as they gain a more comprehensive and multi-dimensional view of the phenomena. GENE [11] generated user networks conditioned on the joint representation of users and entities and predicted early controversy of the hot events. Chunlin Li et al. [9] proposed an opinion community detection model by considering the content similarity and the topology structure.

Automatic controversy analysis poses great challenges. On the one hand, users demonstrate a variety of interactions on social networks, such as commenting, liking, and retweeting content. These interactions foster increased discussion and exchanges, potentially culminating in conflicts and event controversies. Moreover, different topics can have varying effects on controversy: topics attracting high public attention are characterized by rapid and widespread information dissemination. Once distinct opposing camps are formed, an event with high public attention may escalate into a controversy. However, the majority of existing controversy analyses [10] consider only one type of interaction relationship and construct homogeneous networks, which leads to the loss of information. Most importantly, different attributes can trigger different levels of controversy in social networks. For example, users with different genders or social statuses may hold different perspectives on the same event. Previous studies have largely neglected the integration of richer information and the provision of more user attribute details, despite their significance in controversy analysis. In addition, user-generated information often conveys user’s sentiment, which is also an important feature need to be recognized in controversy detection [12]. However, the existing studies mainly focus on the explicit content and relationships within the hot event conversation. They cannot detect the existence of subtle information communication, which greatly limits the understanding of polarity. To overcome the above limitations, the primary objective of this paper is to explore how to better exploit and integrate richer intrinsic text features, topological features, sentiment features, and attributed features for controversy analysis. So, we constructed a novel research framework based on a heterogeneous signed attributed network to investigate the problem of quantifying controversy, which increases the power of information fusion in the task of controversy detection.

In this paper, a framework based on a heterogeneous signed attributed network and deep dual-layer self-supervised community analysis is proposed to fully characterize the relationship network of hot events discussion on social media. Our proposed framework creates a comprehensive and rich representation of the hot events discussions. Moreover, the model offers a comprehensive portrayal of hot events, capturing a wealth of information regarding user interaction relationships, attributes, sentiment, comments, summaries and topics. Due to previous studies proving that controversial events exhibit a community structure [1], we can perform community detection based on the constructed hot event network. Furthermore, we evaluated the level of controversy for a hot event from three perspectives: community connection, communication, and representation. This approach aims to enhance the reliability of the results and derive a final controversy score. The community results were verified to be significant under the evaluation of *p*-value scores for each community. To the best of our knowledge, it is the first paper to employ heterogeneous signed attributed network to compute controversy. Additionally, we developed an online service platform to facilitate users for the analysis of other hot events, which is available at http://www.csbg-jlu.site/contro/, accessed on 7 February 2025.

Our approach brings some key innovations with respect to previous research on controversy analysis. In summary, we highlight the main contributions as the following four aspects:

- We propose a novel framework based on a heterogeneous signed attributed network to quantify controversy, which thoroughly and comprehensively integrates all aspects on social networks. We also attempt to explain why an event is controversial or not;
- Multidimensional semantic, structural and attribute information are incorporated into the heterogeneous signed attributed network;
- The quantified result integrates three measures from the perspectives of network connectivity, communication, and network representation;
- An online service platform for controversy analysis of hot events has been developed, providing open data and source code for users’ convenience.

## 2. Related Work

With the emergence of the Internet and social media, the field of controversy research has entered a new era. Currently, researchers are increasingly focusing on technical tools such as big data analysis, sentiment analysis, and social network analysis to conduct controversy detection. Table 1 summarizes the related work categorized along different dimensions.

### 2.1. Content-Based Approaches

Content-based approaches only consider text content produced by users in a hot event discussion. Early scholars, primarily sociologists, studied controversies by evaluating user content, conducting various surveys, or analyzing data based on statistical methods [1,13]. This method represents a traditional approach to studying controversies. Cantador [14] applied these techniques to analyze recorded citizen participation in a real e-participatory platform, integrating thematic and geographical metadata. Their focus was on analyzing controversy to better understand the complexities of the proposals and debates raised from citizen participation. With the development of natural language processing (NLP) technology, controversy analysis leverages a range of techniques, including sentiment analysis and topic detection. The most work in this aspect involves extracting and assigning scores for controversial words and phrases, such as political terms, sensitive topics, or emotionally charged language. By examining the occurrence of controversial words and evaluating the similarity of sentences, researchers can gain insights into the level of controversy of a hot event. Ril et al. [4] detected controversial political topics based on semantic analysis of text and proved their usefulness in finding and quantifying controversial topics. Ignatow [15] found topics of news articles and analyzed them based on sentiment analysis models. Choi [16] assigned sentiment scores to articles based on their text content and extracted topics and subtopics.

The rapid development of social media platforms has provided researchers with a vast amount of data resources for analyzing the controversial characteristics of hot events. They enable more people to engage in discussions and debates on controversial hot events. Popescu et al. [17] analyzed and extracted features and patterns from Twitter to identify and discern controversial content. Beelen et al. [18] utilized comments on articles to identify their extent of controversy. They extracted four sets of features, including structural, linguistic, emotional, and content similarity. Random Forest and Support Vector Machine algorithms were employed to predict the controversial level of news.

### 2.2. Network-Based Approaches

The network-based method focuses on discovering the controversial nature of hot events from the topological relations of the network. It aims to overcome the limitations of a content-based approach that is influenced by cultural context and professional knowledge. Duoqi Song [19] specifically focused on the structural patterns of negative citations, impact assortativity of involved papers and the consequences of receiving negative attention. Previous research [20] has demonstrated the existence of a network structure of adversarial communities for a controversial hot event. And, the controversial extent of the hot event is quantified by evaluating the features of communities, such as Random Walk and betweenness controversy score, which are context-free and easy to use. The accuracy and performance of opinion community detection were improved significantly over network-based models. Numerous studies have proposed methods to analyze conversation graphs on Twitter [16,21]. For instance, Guerra et al. [3] were the first to use network structure to measure controversy by quantifying polarization in the network. Based on the community detection algorithm, the network was divided into two distinct, non-overlapping communities. They showed that the polarized event has a low density of high-degree nodes in the boundary set. Qiu et al. [7] developed an emotional social network model that takes into account the strength of users’ sentiment. The authors divided the network into communities with different sentiments by a simulated annealing algorithm, and assessed controversy based on sentiment differences. To measure a controversial event based on the separated graph network, Garimella et al. [22] built conversation graphs in different ways, including retweet graph, content graph, and hashtag graph. Research by Bail et al. [1] revealed that the exposure of social media users to opposing views can increase political polarization. Moreover, this effect is found to be influenced by the social relationships of the users. Thus, opinion leaders are also an important factor in controversy analysis. Matakos et al. [23] defined a novel polarization index for quantifying polarization in a network, based on the opinions of users under a popular opinion formation model. However, these traditional graph divisions cannot extract higher-level features in the network. Coletto et al. [24] proposed a motif method for finding controversial issues, which conducts its results only based on patterns of network structure.

### 2.3. Combined Model Approaches

The combined model integrates content and network to measure controversy. Originally, the researchers [8] focused on the specific structure of the conversation tree and predicted early controversy based on the discussion threads, which included comments and retweets. The study demonstrated that higher-quality textual representations for conversation trees could boost predictive performance. Therefore, some deep learning methods are introduced to obtain valuable semantic information in later studies. In particular, word embedding technologies like Word2vec [25] and BERT [26] have demonstrated excellent semantic features for numerous downstream tasks and have made textual features more robust and less brittle. Researchers applied the deep semantic information mined to a combined model for controversy analysis. Moreover, the development of graph neural network (GNN) has greatly contributed to the advancement of controversy analysis, which can fuse semantic features and network structure to infer user polarity. In the case of GNN, various network models are applied to construct social networks to study the controversy of hot events. Samy Benslimane et al. [27] considered controversy detection as a classification task. They adopted state-of-the-art GNN techniques in conjunction with machine learning-based natural language processing to integrate both structural information and discussion contents. Mendoza et al. [11] generated user networks conditioned on the different entities to analyze comments information on multiple relationships. They showed that the improved representation of the network helps detect polarized communities. To better model sentiment information in controversy analysis, some novel network algorithms have emerged. Bonchi et al. [28] made an eigenvector-based method for computing controversy in signed graphs. This approach characterizes sentiment text information as signed edges to construct a signed network. Hohmann et al. [29] proposed a method to quantify ideological polarization using the generalized Euclidean distance, which estimates the difficulty of traversing a network from one opinion to another, taking into account both opinion divergence and community structure. Muñoz et al. [30] proposed an SPIN algorithm which quantifies political polarization in online social networks by analyzing the flow of negative information between and within communities, leveraging network structure and information theory principles. The algorithm proposed by Chang Guo et al. [31] innovatively combines the physical field model with the analysis of multi-scale propagation intensity. While effectively integrating the structural information of heterogeneous networks, it gives particular consideration to the core element of node influence. Yan Zhao et al. [32] combined the semantic graphs generated by multi-source meta-paths and took it as the objective of structural reconstruction to achieve unsupervised community detection.

**Table 1:** The related work of controversy analysis.

Type	Method	Network	Content	Sentiment	Web
Content-based	Concept-level sentiment analysis [4]	-	words and topic	polarity lexicons	-
	Topic sentiment analysis [15]	-	topic and sentiment	sentiment- polarity index	-
	Links to controversial article [16]	-	article and topic	-	-
	Heuristic fine-grained measurement [33]	-	offensiveness topic	sentiment	-
	Automatic controversy analysis [14]	-	comment and topic	-	https://madrid4u.es/planes-en-madrid, accessed on 7 February 2025
Network-based	Polarization index [23]	User’s opinion graph	-	computing user opinion	
	Reconstruct complete conversation [3]	Conversation graphs	-	-	
	Random Walk [22]	Content graph	-	-	
	Observer asymmetric polarization [1]	Directed network of electors	-	-	
	Investigating Opinion Distribution [7]	Emotional social network	-	edge connection frequency	
Combined model	Generation named entities [11]	Multi-relation graph	title and comments		-
	TPC-GCN [12]	TPC DTPC	topic post comment	-	-
	Opinion community detection [9]	User network	user opinion	emotional tendencies of the words	-
	Early detection approach [8]	Conversation thread trees	post	-	-
	POST + C-Text Rate Tree [27]	User post network	post	-	-

## 3. Method

**Figure 1:**
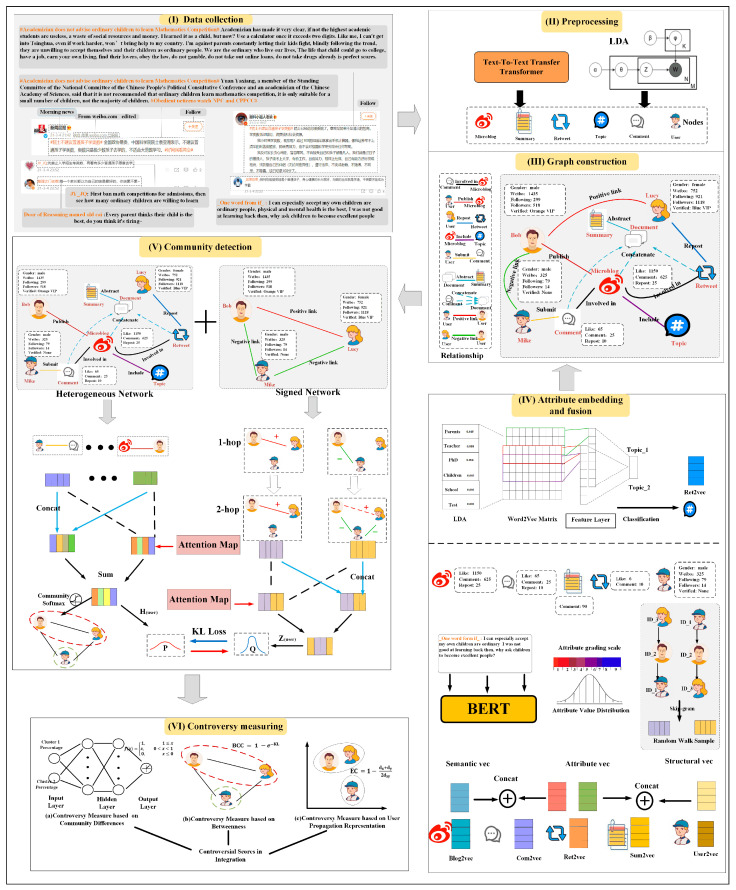
Controversy analysis framework: the whole framework consists of six modules, namely (I) data collection, (II) preprocessing, (III) graph construction, (IV) attribute embedding and fusion, (V) community detection, (VI) controversy measuring.

The overall framework of our proposed controversy analysis, as illustrated in Figure 1, primarily consists of six modules as follows: data collection and dataset description, preprocessing, heterogeneous signed attributed network construction, attribute embedding and fusion, community detection, and controversy measures. Subsequently, detailed descriptions of these modules will be provided.

### 3.1. Data Collection and Dataset Description

We collected data from Weibo (https://weibo.com/, accessed on 7 February 2025), which is a Twitter-like social media platform in China. Netizens actively engage in a diverse array of discussions on Weibo, covering a wide range of hot events that extend beyond national issues to everyday life. Users can also follow other users, actively participate in discussions by commenting on and liking posts, and share interesting content with their followers. Weibo provides a dynamic and interactive social media experience, connecting millions of users and fostering communication, expression, and engagement within the Chinese online community. As a result, multiple social relationships are formed, contributing to a thriving online social ecosystem.

We selected five hot events from Weibo between January 2017 and October 2022, which include the Mathematical Olympiad, the Double reduction policy, heavy rain in Shanxi, the Luding earthquake, and Sexy Tea. We crawled comprehensive discussion information from Weibo, which included original microblogs, comments, retweets, and involved users. The attribute information was also extracted, including user attributes such as their gender, the number of microblogs they have posted, the number of followers, the number of followings, and their account status, as well as microblog attributes such as the number of likes, comments, and retweets as node attributes. To enhance data utilization and sharing, we utilized the DeepL translator to translate the data into English. The background descriptions of the five hot events as follows:

**Mathematical Olympiad:** During the Two Sessions in 2021, Yuan Yaxiang, a member of the Standing Committee of the National Committee of the Chinese People’s Political Consultative Conference and an academician of the Chinese Academy of Sciences, proposed that ordinary children should not be required to learn Olympiad mathematics. This suggestion sparked widespread debate in society;

**Double reduction policy:** The “double-reduction” policy, introduced by China’s Ministry of Education in 2021, sparked intense debate. This policy aims to ease the burden of excessive homework and off-campus tutoring on students, promoting a healthier education environment. While many praised it for fostering a more balanced approach to learning, some expressed concerns about potential impacts on academic performance;

**Heavy rain in Shanxi:** Torrential rain downpours in Shanxi in October 2021 affected the lives of local people. Rescue teams coordinated their efforts to save those trapped people. On social media, people spontaneously organized themselves and actively participated in disaster relief efforts through voluntary participation, donations, and other means. Netizens extended their encouragement to the people of Shanxi province, expressing hopes for their swift recovery from the difficulties they are facing;

**Luding earthquake:** In 2022, a series of earthquakes struck Luding County in Ganzi Prefecture, Sichuan Province, China, with the most severe being a magnitude 6.8 earthquake on 5th September. The earthquake caused significant casualties and property damage. Following the earthquake, public opinion was highly concerned. On the one hand, people expressed deep sympathy and concern for the destruction and casualties caused by the earthquake, offering condolences and support through various channels. On the other hand, the progress and effectiveness of rescue efforts were also closely watched by the public;

**Sexy Tea:** In the autumn of 2022, the popular Chinese tea brand Teayan found itself embroiled in a significant controversy when two of its outlets in Nanjing renamed their shop signs to “SexyTea”, deviating from the original name “茶颜悦色” (which translates to “charming tea color” or “pleasing tea appearance”). This unprecedented move triggered a firestorm of debate and criticism online.

The statistics of the dataset are shown in Table 2.

### 3.2. Preprocessing

This process aims at extracting the different entities and interaction of the discussion for the preparation of graph construction. We collected data on original microblogs, their retweets, and comments, and filtered out records where the sum of retweets and comments was less than 5 to ensure that the selected users had sufficient interaction activity. While identifying existing information such as user nodes, microblog nodes, comment nodes, retweet nodes, and their relationships, we used text mining techniques to extract “topic” and “summary” nodes. During the preprocessing stage, we performed word segmentation, stopword removal, and tokenization. Based on these two models, “topic” and “summary” node type are introduced to represent the focal information of the discussion.

**Figure 2:**
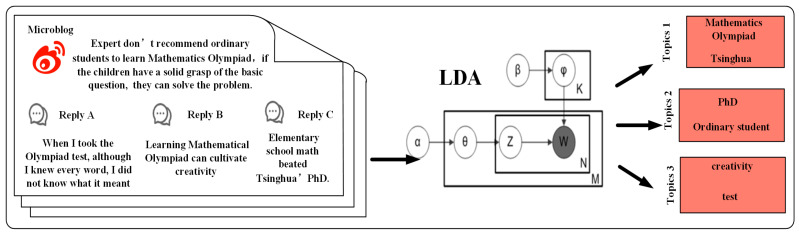
Topic detection of a hot event.

Topic models are widely used to infer the underlying topics from the observed words in the documents. One of the most commonly used topic models is Latent Dirichlet Allocation (LDA) [34], which assumes that documents are distributions over topics, and topics are distributions over words. Here, we use the entire discussion of the event as input corpus and utilize topic modeling to assist in the task of community detection. Precisely, the entire document is treated as an unordered word sequence, and guided by the probability model, we derived distributions of topics and words. We identified the topics and selected feature words with the highest probability for each topic. The topic model is illustrated in Figure 2.

**Figure 3:**
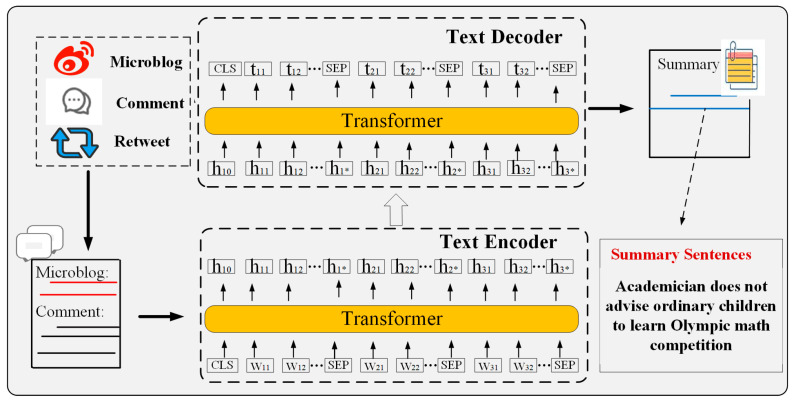
Text summarization based on Text-to-Text Transfer Transformer.

For the method of generating summaries of social media events, we utilized a generative summarization framework. By integrating the original Weibo texts, user comments, and retweeted content, we formed a complete corpus, thus solving the problem of semantic sparsity of a single piece of social media information. In terms of the model, we adopted the end-to-end pre-trained architecture of the Text-to-Text Transfer Transformer (T5) [35]. With the aid of the encoder–decoder structure, we achieved the generation transformation from the integrated corpus to the summary. The system process is illustrated in Figure 3, specifically encompassing the cleaning and alignment of multi-source data, semantic encoding with context, and summary generation.

### 3.3. Heterogeneous Signed Attributed Network Construction

In this section, we formulate the problem of the heterogeneous information network construction. For better illustration, we split the heterogeneous network into the heterogeneous attributed network and signed network.

**Heterogeneous attributed network.** The *heterogeneous attributed network* is denoted as $G_{h}=(V,E,A)$, where:

- V is a set of nodes, which can be further divided into multiple disjointed sets based on node types, V = $\;\cup_{t\in T}V_{t}$, where T is the set of node types and $V_{t}$ is the set of nodes of type t. Six node types are introduced in this paper, including user, microblog, comment, retweet, topic, and summary;
- E is a set of edges, which can also be divided into multiple sets based on edge types, $E=\cup_{r\in R}E_{r}$, where R is the set of relationship types and $E_{r}$ is the set of edges of type r. The network includes six heterogeneous relationships and two signed relationships between users. The user–microblog relationship represents that users publish microblogs. The user–comment and user–retweet represent the two behaviors of commenting and retweeting on the microblog. To establish associations between comments and microblog nodes, we introduce the comment–microblog relationship. For topic nodes, a microblog is assigned to a topic when the cumulative probability of its words matching the topic’s distribution exceeds a predefined threshold δ, forming microblog–topic associations. To derive a concise event representation, three core topics are extracted for each event. Furthermore, based on the extracted summary rules, we introduce a virtual document node to merge microblogs, comments, and retweets. The content of these virtual documents serves as input for text summarization, generating summary nodes that encapsulate textual information. Connections are established between summary nodes and their corresponding microblogs. Additionally, to enrich the connection relationships, we utilized the BERT [26] model to obtain vector representations of the summaries, and based on cosine similarity, established summary–summary when the similarity between summary nodes exceeds the threshold ρ;
- A is a function that maps each node to its attribute vector, $A:V\to R^{d}$, where d is the dimensionality of the attribute space. For user profile, we introduce the individual attributes of users, including their gender, the number of microblogs they have published, the number of followers, the number of followings, and their account status. For the original microblog, we incorporate the number of likes, comments, and retweets as node attributes in addition to the textual information of the microblog. For its comments and retweets, we select the node’s own text messages and the number of likes as node attributes. Furthermore, we directly utilized the summary text as the attribute of the summary node and used the set of words describing the topic as the attribute of the topic.

In addition, a heterogeneous attributed network includes a type mapping function ∅:V→T that assigns a type to each node and a relation mapping function ψ:E→R that assigns a type to each node.

**Signed network**. The *signed network* is a type of social network where the relationships between nodes are characterized not only by their presence but also by the sign of the relationship (positive or negative). Formally, a signed network is denoted as $G_{s}=(V,E,s)$, where:

- V is a set of nodes, representing the set of users who are involved in the event discussion;
- E is a set of edges, representing the relationships between pairs of nodes. If e=(i,j) is an edge in E, it means that nodes i and j are connected;
- In a signed network, the edge sign function s:E→{−1,+1} is used to represent the emotional relationship between users (+1 for a positive relationship and −1 for a negative one). We established signed relationships by analyzing the sentiment of user comments. Here, $s_{ij}=+1$ indicates that user i has a positive attitude towards user j, while $s_{ij}=-1$ indicates a negative attitude.

### 3.4. Attribute Embedding and Fusion

In this section, we will perform attribute embedding and fusion. To create a comprehensive attributed description of a node, we integrated different attribute information into a unified node feature. For nodes that contain text attribute information, we obtained the vector representation of the nodes using the BERT [26] model. We aimed to facilitate the fusion of text attributes with extracted attributes. Cai Desheng [36] et al. fused demographic attributes (e.g., age, gender, the number of clicks) with textual attributes through concatenation to better understand user preferences and intent, achieving improved product recommendations. It is important to note that this attribute information may be affected by attribute imbalance. Specifically, influential users typically have far more followers than ordinary users. Yet, in the context of a hot event, ordinary users comprise the majority of participants. To solve this problem, we categorized the attributes into ten grades based on their distribution and assigned each attribute to the corresponding grade based on its specific attribute value. In addition, to capture the social relationship of user nodes in the network, the node2vec [37] algorithm was used to obtain the user node vector. For the topic node, it is defined by the words with the highest probability. The traditional methods to represent its features involve concatenating or summing the word2vec. It is difficult to capture word-level granularity with this simple processing way. Taking inspiration from the TextCNN algorithm [38], we stacked the word2vecs of each topic into a word vector matrix by the probability of words. Subsequently, we utilized different convolutional kernels to extract new features. By classifying these features, we can determine the corresponding topics. The results of the feature layer were used as topic features after several rounds of training. This process is as illustrated by the attribute fusion module in Figure 1. As a result, we merged the information from the nodes by concatenating their respective vectors into a unified feature vector.

### 3.5. Dual-Layer Self-Supervised Community Detection Based on a Heterogeneous Signed Attributed Network

The architecture of the proposed community detection model is shown in Figure 1. It is a dual-layer self-supervised framework, which can assign users to opposing communities. We first used sentiment analysis to construct a signed network based on the raw network. Then, we input multi-representations into the heterogeneous network and signed network, respectively. We incorporated the attention mechanism in the message propagation process to capture both global and local relationships. By applying softmax to the output of the last layer of the heterogeneous network, we can obtain community memberships. Additionally, we designed a dual-layer self-supervised mechanism to train both the signed and heterogeneous networks. In the following subsections, we will introduce community detection in detail.

#### 3.5.1. Signed Network Extraction

Signed networks are complex networks in which edges have positive or negative properties. It is a useful tool to model the sentiment relationships among users, as it allows for the representation of positive and negative edges that indicate trust, like, praise, or distrust, dislike, and blame in real social relationships. We can build edges between users when one user comments or retweets another user’s microblog. The sentiment analysis determines the attributes of the edges; positive sentiment is connected with “+” edges, while negative sentiment is connected with “−” edges. We construct the user signed network-based on this rule. We developed a website that provides sentiment analysis functionality, which was trained using a large amount of data with a BiLSTM model.

#### 3.5.2. Heterogeneous Attributed Network Interaction Layer

As we know, different types of neighboring nodes of one node capture different information in heterogeneous networks. However, GCN is a neural network that operates on a homogeneous graph based on their neighborhood nodes. Our network was heterogeneous, that cannot be directly applied to GCN. To address the problem, we considered aggregating nodes under different types separately, which projects their features into common space with respective transformation matrix:

(1) $$H^{\left(l+1\right)}=\sigma \left(\sum_{\tau \in T}{\tilde{A}}_{\tau }\;\cdot H_{\tau }^{\left(l\right)}\;\cdot {\;W\;}_{\tau }^{\left(l\right)}\right)\;$$

where  σ is the activation function that considers non-linearity and improves the expressiveness of the model. ${\tilde{A}}_{\tau }$ is an adjacency matrix whose rows represent the nodes in a type and the columns represent the nodes in another type, τ  is the relationship between these two nodes. The node vector representation of type τ at layer 0 corresponds to the original node features, represented as: $H_{\tau }^{\left(0\right)}=X_{\tau }$. $W_{\tau }^{\left(l\right)}$ is a projection matrix that transforms the features into another space. Since different neighbor nodes can have different effects, based on the above method, we considered the importance of attention mechanisms. In addition, there are multiple relationships in the heterogeneous network, we also considered the type-level attention. First, we introduce the neighborhood attention.

(2) $$e_{ij}^{\tau }=a(W_{a}h_{i},\;W_{b}h_{j})$$

(3) $$\alpha_{ij}^{\tau }=softmax(e_{ij}^{\tau })=\frac{exp(e_{ij}^{\tau }\;)}{\sum_{k\in N_{i}^{\tau }}exp(e_{ik}^{\tau })}$$

The attention of a neighbor node is applied to each node under *τ* type by a parameterized matrix. Equation (2) indicates the importance of node j’s feature to node i’s feature. To make coefficients easily comparable among different types of nodes under different types, we used the softmax function to normalize the attention scores. Equation (3) shows the detail of normalization. We represent the embedding of the type τ as $h_{i}^{\tau }=\sum_{v\prime }{\tilde{A}}_{vv\prime }^{\tau }h_{v}^{\prime }$, which is attention’s sum of a neighboring node’s feature.$\;{\tilde{A}}_{vv\prime }^{\tau }\;$represents the attention matrix of τ type. In the next step, we calculated the type-level attention based current embedding as follows:

(4) $$b_{\tau }=\sigma (\mu_{\tau }^{T}\;\cdot \;h_{\tau }\;)$$

(5) $$\beta_{\tau }=\frac{exp(b_{\tau }\;)}{\sum_{\tau \prime \in T}exp(b_{\tau \prime }\;)}$$

where $\mu_{\tau }^{T}$ is an attention vector for the τ type, σ is an activation function. We normalized all τ types using Equation (5). As shown in Equation (6), the representations of different types are summed according to the attention coefficients. The node representation of attention in a heterogeneous network could be obtained in the following:

(6) $$h_{i}^{att}=\sum_{\tau \in T}\beta_{\tau }\;\cdot \;h_{i}^{\tau }$$

To further increase the reliability of the model, we simply concatenated without using type-level attention. Equation (7) shows the concatenation of different representations. The feature space is projected into the initial dimension by Equation (8).

(7) $$h_{i\prime }^{cat}=Concat(h_{1}^{\tau }\;||h_{1}^{\tau }\cdots ||h_{T}^{\tau }\;)$$

(8) $$h_{i}^{cat}=h_{i\prime }^{cat}\;\cdot \;W_{c}$$

(9) $$h_{i}=sum(h_{i}^{cat},\;h_{i}^{att})$$

After the attention mechanism and concatenation process, the embedding of users in heterogeneous network is obtained by Equation (9).

#### 3.5.3. Signed Network Interaction Layer

**Figure 4:**
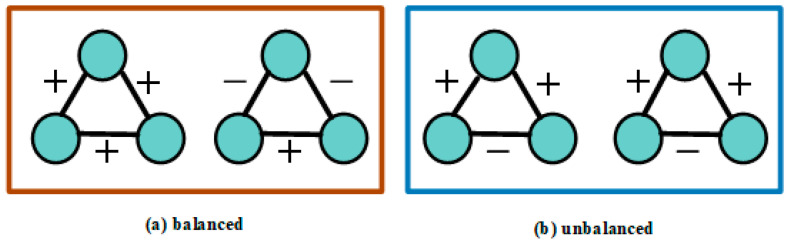
The types of triangles in a signed network.

As illustrated in Figure 4, the balance theory implies “the friend of my friend is my friend” and “the foe of my friend is my foe”. In a signed network, the message passing needs to consider balance and unbalance situations, which is different from heterogeneous network. According to the balance theory, the user nodes are divided into balanced and unbalanced sets as follows:

(10) $$\left\{\begin{array}{c} B_{i}\left(1\right)=\left\{u_{j}|u_{j}\in \;N_{i}^{+}\right\};\;U_{i}\left(1\right)=\left\{u_{j}\right|u_{j}\;\in \;N_{i}^{-}\}\;if\;l=1\\ B_{i}\left(l+1\right)=\left\{u_{j}|u_{k}\in B_{i}\left(l\right)\;and\;u_{j}\in N_{k}^{+}\right\}\;\cup \left\{u_{j}|u_{k}\in \;U_{i}\left(l\right)\;and\;u_{j}\in N_{k}^{-}\right\}\;\\ U_{i}\left(l+1\right)=\left\{u_{j}|u_{k}\;\in \;U_{i}\left(l\right)\;and\;u_{j}\in \;N_{k}^{+}\right\}\cup \left\{u_{j}|u_{k}\in B_{i}\left(l\right)\;and\;u_{j}\in N_{k}^{-}\right\}\;if\;l>1 \end{array}\right.$$

where ${\;N\;}_{k}^{+}$ represents the positive neighbors of $u_{k}$, $B_{i}\left(l+1\right)$ and ${\;U}_{i}\left(l+1\right)$ represent balanced sets and unbalanced sets with length $\left(l+1\right)$, respectively. Next, the neighborhood information is aggregated from different sets.

(11) $$h_{i}^{B(1)}=\sigma (W^{B\left(1\right)}\left[\sum_{j\in N_{i}^{+}}\frac{h_{j}^{\left(0\right)}}{\left|N_{i}^{+}\right|}\;||\;h_{i}^{0}\right])$$

(12) $$h_{i}^{u(1)}=\sigma (W^{u\left(1\right)}\left[\sum_{j\in N_{i}^{-}}\frac{h_{j}^{\left(0\right)}}{\left|N_{i}^{-}\right|}\;||\;h_{i}^{0}\right])$$

Equations (11) and (12) directly utilize $B_{i}\left(l\;\right)$ and ${\;U}_{i}(l)$ to aggregate information, where $W^{U\left(l\right)}$, $W^{B\left(l\right)}$ are transferred matrix. For the aggregation layers for  l>1, we use the following function:

(13) $$h_{i}^{B(l)}=\sigma (W^{B\left(l\right)}\left[\sum_{j\in N_{i}^{+}}\frac{h_{j}^{B\left(l-1\right)}}{\left|N_{i}^{+}\right|}\;||\;\sum_{k\in N_{i}^{-}}\frac{h_{k}^{U\left(l-1\right)}}{\left|N_{i}^{-}\right|}\;||\;h_{i}^{B(l-1)}\right])$$

(14) $$h_{i}^{U(l)}=\sigma (W^{U\left(l\right)}\left[\sum_{j\in N_{i}^{+}}\frac{h_{j}^{U\left(l-1\right)}}{\left|N_{i}^{+}\right|}\;||\;\sum_{k\in N_{i}^{-}}\frac{h_{k}^{B\left(l-1\right)}}{\left|N_{i}^{-}\right|}\;||\;h_{i}^{U(l-1)}\right])$$

(15) $$h_{i}^{sign}=Concat(h_{i}^{B(l)}||\;h_{i}^{U(l)}\;||\;h_{i}^{\left(l\right)})$$

We utilized Equations (13) and (14) to derive the representation of the user nodes under balanced and unbalanced conditions. Finally, we updated the users’ representation by fusing three information sources: the user self-representation from last layer, information from negative neighbors, and information from positive neighbors. The signed representations of user nodes are obtained using Equation (15).

#### 3.5.4. Self-Supervised Module

After the above dual-layers modules, we obtained the multi-representation of the user node in two different ways. In order to complete the community detection, we need to unify these two modules into a uniform framework. Here, for the i-th user in the j-th community, we used the Student’s t-distribution as a kernel to evaluate the similarity between the representation $h_{i}$ from a signed network and the cluster center vector $u_{i}$ as follows:

(16) $$q_{ij}=\frac{{\left(1+{\left||h_{i}-u_{j}|\right|}^{2}/v\right)}^{-\frac{v+1}{2}}}{\sum_{j\prime }{\left(1+{\left||h_{i}-u_{j\prime }|\right|}^{2}/2\right)}^{-\frac{v+1}{2}}}$$

where $h_{i}$ is the i-th user’s representation from signed network. The K-means method is utilized to initialize $u_{j}$. v  is degrees of freedom. $q_{ij}$ can be considered as the probability of assigning the i-th user to the j-th community. According to Q distribution, we optimized the representation by a high confidence assignment, which can improve community cohesiveness. The target distribution is calculated as follows:

(17) $$p_{ij}=\frac{q_{ij}^{2}/f_{j}}{\sum_{j\prime }q_{ij\prime }^{2}/f_{j\prime }}$$

(18) $$L_{sign}=KL\left(P||Q\right)=\sum_{i}\sum_{j}p_{ij}log\frac{p_{ij}}{q_{ij}}$$

where $f_{j}=\sum_{i}q_{ij}$ is the soft assignment frequency. Equation (18) indicates that minimizing the KL divergences loss will contribute to learning a better representation for community detection task. P  distribution and Q  distribution are updated to complete the self-supervised learning in turn.

As for the representation in heterogeneous modules, it is possible to transform them to a Z distribution as the truth labels with a softmax function. We use the  Z distribution to guide the community discovery tasks. As is mentioned before, it is feasible to utilize P distribution to supervise Z distribution, and complete the unification of two modules as follows:

(19) $$Z=softmax(h_{i}^{att}\cdot W^{d})$$

(20) $$L_{dual}=KL\left(P||Z\right)=\sum_{i}\sum_{j}p_{ij}log\frac{p_{ij}}{z_{ij}}$$

In the community detection, the calculation of the community of each user node depends on nodes that interact with them in heterogeneous networks. After finishing the calculation, all users will be divided into different communities.

### 3.6. Controversy Measure Integration

We quantified the extent of controversy of the hot event based on the result of community detection. The aim of controversy measure is to capture how separated the two communities are. We utilized three measurement methods as follows: based on community difference, based on betweenness, based on user propagation representation. Details are shown in the following.

#### 3.6.1. Controversy Measure Based on Community Difference

Proportional differences in communities for controversial hot events are small. In order to obtain controversy value, we made an assumption that the controversy value is the largest when two polar communities are equal. The feed forward network consists of an input layer, hidden layer, and an output layer. We introduced a forward neural network to predict the extent of controversy. Qiu Lin et al. [7] manually assigned controversy value in order to construct a training set. They obtained model parameters for controversy measures after a lot of training. Here, we utilized the completed trained feed forward network model for controversy measure.

As the controversy measure module is shown in Figure 1, we took the percentage of communities as input to the network model. We abstracted the input data to another dimensional space in a hidden layer. Finally, the output layer employs the activate function to obtain controversy score:

(21) $$f\left(x\right)=\left\{\begin{array}{c} 1,\;1\leq x\\ x,\;0\leq x<1\\ 0,\;otherwise \end{array}\right.$$

where Equation (21) represents an activate function which maps the result to a real number from zero to one. We assigned the value close to zero when controversy is small, and close to one when the controversy value is large.

#### 3.6.2. Controversy Measure Based on Betweenness Centrality

Betweenness centrality is an important indicator for measuring the information control ability of network nodes. Its value is obtained by calculating the proportion of the shortest paths passing through this node to the shortest paths of all node pairs. The higher the value, the more significant the bridging role of this node in connecting different groups. The function of betweenness centrality bc(e) is listed as:

(22) $$bc\left(e\right)=\sum_{s\neq t\in V}\frac{\sigma_{s,t(e)}}{\sigma_{s,t}}$$

where $\sigma_{s,t}$ is the total number of shortest paths between nodes s, t. $\sigma_{s,t(e)}$ is the number of the shortest paths which include edge e. Structural holes in well-separated communities imply that bridging nodes exhibit high betweenness centrality. Consequently, betweenness centrality values across distinct communities show high similarity. Conversely, strong inter-community connections reduce this similarity, indicating poorer separation. Based on this relationship, we define the betweenness centrality controversy (BCC) metric in Equation (23):

(23) $$BCC=1-e^{-d_{KL}}\;$$

where $d_{KL}$ denotes the KL divergence about two communities, which is obtained by computing KL divergence of the betweenness centrality. In the BCC function, we can obtain a controversial community measure result, BCC∈[0, 1].

#### 3.6.3. Controversy Measure Based on User Propagation Representation

We obtained the user representation from the heterogeneous network and signed network in the last layer and fused them by concatenating operation. The distance of representation can reflect community separated extent. Inspired by the Davies–Bouldin (DB) index, we defined the function Equation (24) to measure the controversy:

(24) $$Embedding=1-\frac{d_{X}+d_{Y}}{2d_{XY}}$$

where $d_{X}$ represents the average distance of the nodes in the X community, conducting on the sampled nodes from different communities respectively. In addition, the distance $d_{XY}$, represents the average distance between community X and community Y. The EC ranges from zero to one which corresponds to extent of the controversy, defined as an embedding controversy measure.

The above three methods for measuring controversy are, respectively, based on community differences, network structures, and user communication representations. In order to achieve a more balanced and comprehensive evaluation of controversy, we carried out an averaging process for these three methods.

## 4. Results

Firstly, we compared our proposed method with the existing classical methods, including Random Walk [22], Biased Random Walk (BRW) [39], BCC [22], EC [22], GMCK [3], MBLB [40], and community-based [7]. The baseline methods are illustrated as follows:

- **Random Walk Controversy (RWC)**: This method measures the likelihood of a random user encountering authoritative content from the opposing side in a controversy. It uses random walks on the conversation graph to estimate the probability of staying within the same partition versus crossing to the other side. A higher RWC score indicates a higher degree of controversy;
- **Biased Random Walk (BRW)**: It assigns a finite initial energy to each node, representing its influence, and depletes this energy as the random walk progresses. By focusing on boundary nodes and considering structural and content attributes, BRW measures the ability of ideas to penetrate opposing communities;
- **Betweenness Centrality Controversy (BCC)**: This method analyzes the structural differences between controversial and non-controversial graphs by comparing the betweenness centrality of edges in the cut (separating the partitions) and the rest of the graph. A higher BCC score suggests greater separation between the partitions and a higher degree of controversy;
- **Embedding Controversy (EC)**: This method utilizes low-dimensional embeddings of the conversation graph to measure the separation between the partitions. It calculates the average distance between vertices within and across partitions and uses a measure similar to the Davies–Bouldin index to quantify the level of controversy;
- **GMCK**: This is a boundary controversy measure, named after its four creators: Guerra, Meira, Cardie, and Kleinberg. The measure operates on the premise that in a controversial topic, the boundary nodes are more likely to be strongly linked to the internal node of their respective group rather than to other boundary nodes.
- **MBLB**: This algorithm detects polarization in networks by propagating labels across nodes to form distinct communities. The polarization score (MBLB) ranges from 0 to 1, where 1 indicates perfect polarization (equal-sized, fully divided communities), and 0 represents no polarization. The score decreases when community sizes are unbalanced, reflecting less extreme polarization.
- **Community-based**: Community-based methods calculate controversy by analyzing the structure of the social network constructed from user interactions. They identify communities within the network, where interactions within a community are dense, and interactions between communities are sparse.

The experiments are conducted on the existed controversy detection dataset, including Karate’s Club, US Political Blogs, Gun Control, Brazilian Soccer Teams, NYC teams, University Friendships’ Network [3], and political communication network [41]. The details of the dataset are shown in Table 3.

According to the discussion of controversy thresholds by Shiri Dori-Hacohen et al. [42], we set the threshold for controversy scores at 0.5, defining topics with scores above 0.5 as controversial and those below 0.5 as non-controversial. Table 4 shows the controversy scores on the existing datasets. The text in bold indicates the inconsistencies between the controversy score and the nature of the topic controversy. From the computational results, it can be observed that our calculations align consistently with the controversial nature of the topics. However, the existing baseline algorithms all exhibit discrepancies with the results. This indicates that our method, which takes into account multiple dimensions of attribute feature, possesses greater stability.

In the data we crawled, controversial topics included Mathematical Olympiad, Sexy Tea, and Double Reduction policy, while non-controversial topics included heavy rain in Shanxi and the Luding earthquake. Table 5 displays a comparative analysis of the computational results between existing methods and our proposed method. Bold numbers indicate inconsistencies with the expected results. It can be observed that each method exhibits inconsistencies, but our method consistently yields stable results. The reason behind this lies in the fact that existing methods only consider a specific category or a limited set of features related to controversial issues, whereas our approach integrates the multidimensional features of topics and also takes into account various computational results. Therefore, our proposed method demonstrates strong stability in calculating controversy across different topics. In the following, we further validate and explain the advantages of our proposed model.

In general, the same community views are consistent. Firstly, to verify its significance of the community detection by our model, we computed the p value of communities by using the hypergeometric distribution function in Equation (25). The p-value ranges from 0 to 1, where less than 0.05 usually means statistical significance where the probability of chance occurrence is small. Next, we needed to calculate the sentiment number including positive, negative, and neutral attitudes from the same community. The results can be obtained in Table 6. We derived the p-value by Equation (25):

(25) Pk,N,M,n=Mk∗N−Mn−kNn

where N is the total number of people with dominant emotions, n is the total number of people in all communities, M is the total number of people in a community, and k is the number of people with dominant emotions in a community.

This study is based on heterogeneous graph network modeling to construct a network of hot events with multiple types of nodes and semantic edges. Through the joint analysis of the network structure and node features using deep clustering, the quantification of the controversial nature of the events is achieved. Different from traditional graph partitioning methods that only focus on topological segmentation, this framework explores implicit semantic associations. The generated controversial communities can guide the retrieval module of the Retrieval-Augmented Generation (RAG) system to locate the key evidence of the controversy, avoiding information overload in the open domain retrieval. By combining the heterogeneous network with deep semantic clustering, this study provides new research ideas for the analysis of event controversies and enhances the ability of the RAG system to conduct targeted analysis in complex scenarios.

## 5. Web Server

**Figure 5:**
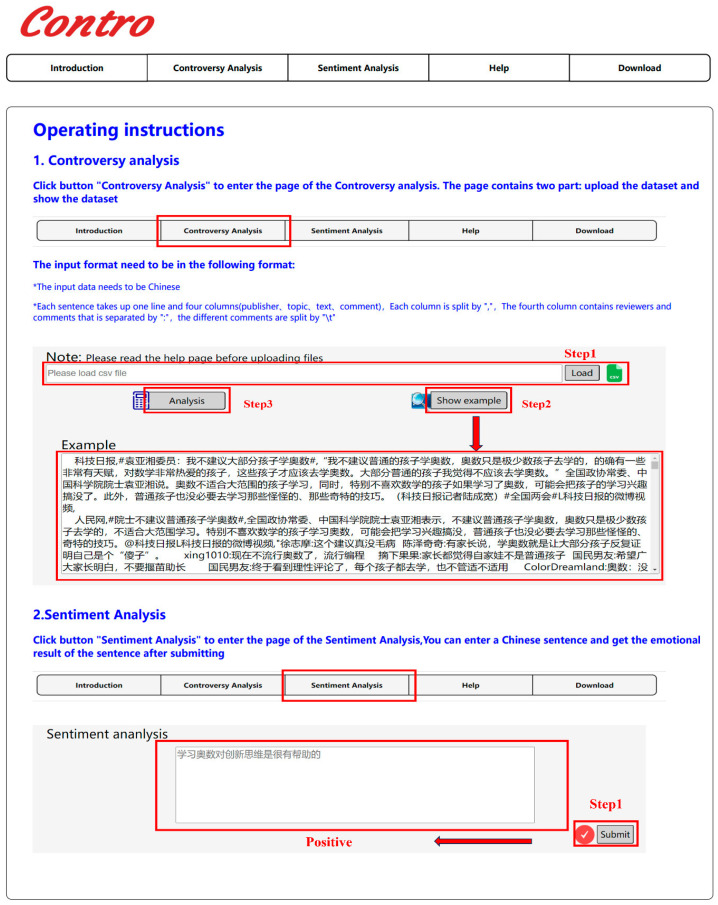
The user guidance of the web server.

We have developed an online web server platform (http://www.csbg-jlu.site/contro/, accessed on 7 February 2025) in order to facilitate users to compute controversy of more hot events. The guidance of the webserver is shown in Figure 5. The website consists of five modules as follows: overview of the proposed algorithm, controversy detection, sentiment analysis, help, and download. Details of each module are introduced as below.

The algorithm’s overview is presented on the homepage, outlining the overall research framework. The core of the website is controversy prediction, and users can input the related textual content of hot events either through a CSV file format or manually. After analysis, the webpage will visually display the controversy report from multiple dimensions, including sentiment distribution, keywords, hot hashtags, and views from opposing groups. We have included a small demo about Mathematical Olympiad as a reference. Based on the experimental results, we can gain a deeper understanding of the intrinsic reasons behind the controversial hot events through comparative analysis and summarization of different viewpoints. Additionally, the web server can predict the sentiment of a Chinese sentence using the sentiment analysis module, which is a crucial step in building the signed network and computing the sentiment distribution. To facilitate user research, the source code and all datasets used in this paper are available on this web server. Moreover, the help page guides users on relevant operations.

## 6. Conclusions, Implications, and Future Work

### 6.1. Conclusions

In this paper, we introduce a novel framework for comprehensively analyzing the controversies of hot events and provide a set of standardized controversial analysis processes by modeling them as heterogeneous signed attributed networks. Different from previous studies that focused on sentence-based evaluations at the micro-level, our approach emphasizes controversy evaluation at the macro-level by integrating heterogeneous information from multiple perspectives, including user attributes, semantic content, and topological structures. The proposed heterogeneous signed attributed network framework innovatively combines symbolic relationships and attribute information to capture the interactions among different entities such as users, comments, and topics. Through the co-training of the heterogeneous subgraph network and the signed subgraph network, the semantics of edges are effectively abstracted while retaining structural and attribute information, thus enabling more accurate community partitioning. The controversy quantification module evaluates the polarization degree of events from three complementary perspectives: community differences, structural betweenness centrality, and user propagation representation. In addition, we have developed an online service platform to facilitate the real-time analysis of controversial events, allowing users to independently explore the community composition, key influencers, and viewpoint distributions.

### 6.2. Theoretical and Practical Implications

This paper has both theoretical and practical implications. At the theoretical level, it the studies of controversy quantification [22,43]. It is the first study situated at integrating the user information, content information, and information propagation structural information on social networks. Previous research on quantifying controversy used limited content information and mainly focused on homogeneous conversation networks on social media, neglecting the influence of user attributes that induce polarization. These research findings are hard to fully reflect the true nature of the topic’s controversy [3]. Different users differ in their contributions to the controversy of an event. For example, the influence of a key opinion leader (KOL) is greater than that of ordinary users, and their difference may be reflected in the number of fans. Thus, neglecting attribute information can have an impact on the results of controversy analysis. In our research, we encompass various types of nodes (users, microblogs, comments, retweets, topics, and summaries), relationships (user–microblog, user–comment, user–retweet, comment–microblog, microblog–topic, summary–summary), and attributes (user profile, microblog attributes, comment attributes, topic attributes) into a heterogeneous signed network. This mechanism allows for a more nuanced understanding of the complex interactions and information flow within a controversy.

Our research also has various practical implications. On the one hand, controversy analysis assists governments, businesses, and organizations in comprehending public sentiment regarding specific events, products, or policies. This, in turn, enables them to promptly address public sentiment, enhance products or policies, and uphold their reputation. On the other hand, we provide a dataset of highly representative hot events for further analysis by other researchers, and we also developed an online webserver tool that incorporates the source code.

### 6.3. Future Work and Discussion

Future research can be expanded in three key directions. First, computationally efficient graph network methods need to be explored to address performance bottlenecks in large-scale event analysis. Second, a multimodal data fusion framework should be developed to integrate unstructured data such as images and videos, thereby enhancing model representation capabilities. Additionally, time-series analysis techniques can be introduced to construct dynamic models that reveal the evolutionary patterns of controversial events. RAG technology, as a prominent research direction, enables productivity enhancement by leveraging real-world data. It is also possible to study graph-enhanced RAG [44] to improve the efficiency of identifying controversial points and the interpretability of controversial analysis. These advancements will further refine the controversial event analysis system, providing more precise technical support for fields such as public opinion monitoring and public policy formulation.

## Figures and Tables

**Table 2:** Statistics of the dataset.

Event	Microblog	Comment	Retweet	User
#Mathematical Olympiad	734	14,642	6212	12,323
#Double reduction policy	228	3067	1716	3673
#Heavy rain in Shanxi	340	6648	10,559	13,150
#Luding earthquake	523	7440	14,512	19,438
#Sexy Tea	1967	13,944	3127	12,743

**Note:** “#” represents a hashtag, which is used for the classification and tagging of content.

**Table 3:** Datasets statistics: media, size of the nodes, and edges.

Dataset	Media	Nodes	Edges	Controversy
#Karate’s Club	Friendships	34	78	Y
#US Political Blogs	blogs	1224	16,715	Y
#Political communication network	Twitter	18,470	48,053	Y
#Gun Control	Twitter	61,740	342,449	Y
#Brazilian Soccer Teams	Twitter	27,415	156,489	Y

**Note:** “#” represents a hashtag, which is used for the classification and tagging of content.

**Table 4:** The controversy scores on the existing datasets.

	Controversy	Random Walk	BRW	BCC	EC	GMCK	MBLB	Our Method
#Karate’s Club	Y	**0.11**	**0.43**	0.64	0.51	**0.17**	**0.11**	0.62
#Political Blog	Y	**0.42**	0.69	0.53	**0.49**	**0.18**	**0.45**	0.65
#Twitter political	Y	0.77	0.81	0.79	0.62	**0.28**	**0.34**	0.53
#Gun Control	Y	0.70	0.66	0.68	0.55	**0.24**	0.81	0.67
#Brazial soccer	Y	0.67	0.74	**0.48**	0.68	**0.17**	0.75	0.55
#NYC teams	N	0.34	0.29	0.24	0.17	0.01	0.19	0.37
#University	N	0.35	0.31	0.26	0.38	0.01	0.27	0.46

**Note:** “#” represents a hashtag, which is used for the classification and tagging of content.

**Table 5:** The comparison on our datasets.

	Controversy	Random Walk	BRW	BCC	EC	GMCK	MBLB	Our Method
#Mathematical Olympiad	Y	0.83	0.69	0.73	**0.45**	0.52	**0.41**	0.79
#Sexy Tea	Y	0.77	**0.44**	0.72	0.68	0.53	**0.38**	0.72
#Double reduction policy	Y	**0.46**	0.72	0.69	0.51	**0.41**	0.55	0.67
#Heavy raining in Shanxi	N	**0.61**	0.38	**0.50**	0.32	0.42	0.39	0.48
#Luding earthquake	N	0.33	0.32	0.29	0.43	0.35	0.44	0.25

**Note:** “#” represents a hashtag, which is used for the classification and tagging of content.

**Table 6:** Community sentiment distribution and *p*-value.

Event	Community	Positive Num	Negative Num	Neutral Num	*p*-Value
#Mathematical Olympiad	community 1	2328	1694	433	6.77 × 10^−16^
	community 2	2077	3021	167	5.33 × 10^−15^
	community 3	608	696	1289	9.43 × 10^−14^
#Double reduction policy	community 1	933	619	88	3.6 × 10^−26^
	community 2	698	1036	72	1.51 × 10^−12^
	community 3	38	34	154	5.97 × 10^−12^
#Sexy Tea	community 1	3468	2104	1537	9.41 × 10^−12^
	community 2	1613	3365	656	5.39 × 10^−25^
#Heavy rain in Shanxi	community 1	5210	2788	863	9.08 × 10^−12^
	community 2	1810	2153	326	6.17 × 10^−9^
#Luding earthquake	community 1	7360	4119	634	1.35 × 10^−22^
	community 2	2706	3811	808	4.11 × 10^−13^

**Note:** “#” represents a hashtag, which is used for the classification and tagging of content.

## Data Availability

The data are contained within the article.
